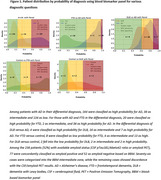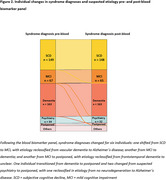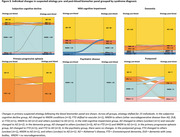# A Prospective Evaluation of Real‐World Experience with an Alzheimer's Blood‐Based Biomarker Panel in Memory Clinics: The CANTATE Project

**DOI:** 10.1002/alz70856_103005

**Published:** 2025-12-25

**Authors:** Sinthujah Vigneswaran, Inge M.W. Verberk, Lynn Boonkamp, Lisanne in ‘t Veld, Rebecca Z. Rousset, Thomas Claessen, Marissa D. Zwan, Rik Ossenkoppele, David H Wilson, Afina W. Lemstra, Yolande A.L. Pijnenburg, Wiesje M. van der Flier, Majon Muller, Harro Seelaar, Charlotte E. Teunissen, Argonde C. van Harten

**Affiliations:** ^1^ Alzheimer Center, Department of Neurology, Amsterdam UMC, Vrije Universiteit Amsterdam, Amsterdam Neuroscience, Amsterdam, Netherlands; ^2^ Neurochemistry Laboratory, Department of Laboratory Medicine, Amsterdam Neuroscience, Amsterdam UMC, Vrije Universiteit Amsterdam, Amsterdam UMC, Amsterdam, the Netherlands., Amsterdam, Netherlands; ^3^ Amsterdam Neuroscience, Neurodegeneration, Amsterdam, Netherlands; ^4^ Neurochemistry Laboratory, Department of Laboratory Medicine, Amsterdam Neuroscience, Vrije Universiteit Amsterdam, Amsterdam UMC, Amsterdam, Netherlands; ^5^ Neurochemistry Laboratory, Department of Laboratory Medicine, Amsterdam UMC, Vrije Universiteit Amsterdam, Amsterdam Neuroscience, Amsterdam, Netherlands; ^6^ Amsterdam Neuroscience, Neurodegeneration, Amsterdam, Noord‐Holland, Netherlands; ^7^ Neurochemistry Laboratory, Department of Laboratory Medicine, Amsterdam Neuroscience, Amsterdam UMC, Vrije Universiteit Amsterdam, Amsterdam UMC, Amsterdam, Netherlands; ^8^ Alzheimer Center Amsterdam, Department of Neurology, Amsterdam UMC, location VUmc, Amsterdam, Netherlands; ^9^ Clinical Memory Research Unit, Department of Clinical Sciences Malmö, Faculty of Medicine, Lund University, Lund, Sweden; ^10^ Quanterix Corp., Billerica, MA, USA; ^11^ Department of Internal Medicine, Geriatric Medicine Section, Amsterdam Cardiovascular Sciences Institute, Vrije Universiteit Amsterdam, Amsterdam UMC, Amsterdam, Netherlands; ^12^ Department of Neurology and Alzheimer Center, Erasmus Medical Center, Rotterdam, Zuid Holland, Netherlands

## Abstract

**Background:**

Blood‐based biomarkers (BBM) are emerging as minimally invasive, scalable and relatively low‐cost options for discriminating different neurodegenerative diseases. Before implementation in clinical practice, it is important to determine their real‐world clinical validity in patients presenting at memory clinics. To prospectively evaluate real‐world experience with a BBM panel, we assessed changes in syndrome diagnosis, suspected etiology and diagnostic confidence after disclosing BBM panel results tailored to common differential diagnostic considerations.

**Methods:**

We included 450 consecutive patients (66 ± 9 years, 38% female, MMSE 25 ± 5) who underwent a standardized diagnostic workup at three academic memory clinics in the Netherlands and provided informed consent. Patients were evaluated at weekly multidisciplinary meetings. BBM panel results (plasma pTau181, GFAP and NfL; Quanterix, USA) were tailored to differential diagnostic considerations (Verberk, Alz&Dem, 2024) and were presented after clinical work‐up, neuropsychological tests and MRI results during the meetings. We evaluated syndrome diagnosis, suspected etiology and confidence in etiological diagnosis before and after disclosing BBM results. Subsequently, the CSF results or amyloid PET findings, if available, were presented.

**Results:**

Among patients with Alzheimer's disease (AD) in their differential diagnosis, 164 (36%) were classified as high probability for AD, 36 (8%) as intermediate, and 134 (30%) as low probability based on the panel (Figure 1). Median diagnostic confidence increased from 80% [IQR = 70–90] to 90% [IQR = 70–90](*p* < 0.001) after BBM results. Confidence increased in 234 patients (52%), decreased in 52 (12%) and remained unchanged in 164 (36%). Syndrome diagnoses were revised in 6 patients (1%) (Figure 2), while suspected primary etiologies were altered in 23 patients (5%): 7 (2%) became unclear, 11 (2%) shifted to AD, 3 (1%) changed from AD to no neurodegeneration, and 2 (<1%) from AD to FTD (Figure 3).

**Conclusion:**

A BBM panel specific to individual differential diagnostic considerations generally led to an increase in diagnostic confidence and in a minority resulted in a different diagnosis. These results suggest that this panel has some added value in daily clinical practice.